# Supplementary material for: The Construction and Exploration of a Comprehensive MicroRNA Centered Regulatory Network in Foxtail Millet (Setaria italica L.)
Source: Front Plant Sci. 2022 May 6;13:848474. doi: 10.3389/fpls.2022.848474 (PMC9121102; doi:10.3389/fpls.2022.848474)
Supplement: Supplementary file 4 [file Data_Sheet_4.zip › Sit-miRN3020_Seita.9G191500.1_623_TPlot.pdf]

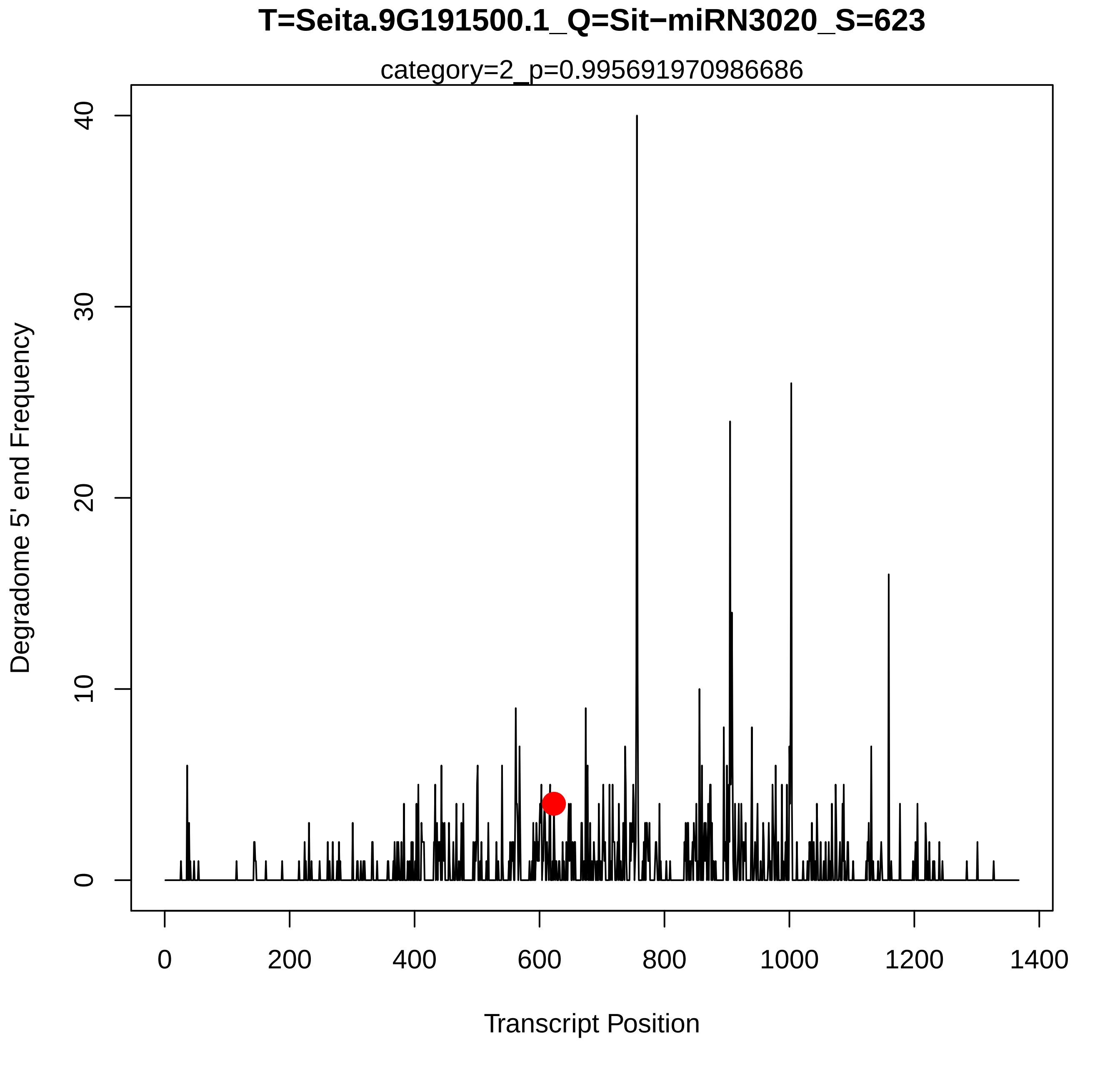

T=Seita.9G191500.1_Q=Sit−miRN3020_S=623
category=2_p=0.995691970986686
Degradome 5' end Frequency
0
10
20
30
40
0
200
400
600
800
1000
1200
1400
Transcript Position
